# The hydroalcoholic extract of *Nasturtium officinale* protectively inhibits apoptotic and inflammatory pathways in hepato- and nephrotoxicity: An *in vivo* study

**DOI:** 10.22038/ajp.2024.25213

**Published:** 2025

**Authors:** Sevil Soudkhah, Sahar Keyghobadi, Amir Shadboorestan, Mahdi Gholami, Behnam Omidi Sarajar, Armin Salek Maghsoudi, Mahmoud Omidi, Saeed Mohammadi Motamed, Saeid Akbarzadeh Kolahi, Nima Rastegar-Pouyani, Shokoufeh Hassani

**Affiliations:** 1 Department of Pharmacology and Toxicology, Faculty of Pharmacy and Pharmaceutical Sciences, Tehran Medical Sciences, Islamic Azad University, Tehran, Iran; 2 Department of Toxicology, Faculty of Medical Sciences, Tarbiat Modares University, Tehran, Iran.; 3 Department of Toxicology and Pharmacology, Faculty of Pharmacy, Tehran University of Medical Sciences (TUMS), Tehran, Iran; 4 Food Health Research Center, Hormozgan University of Medical Sciences, Bandar Abbas, Iran; 5 Department of Pharmacology and Toxicology, Faculty of Pharmacy, Hormozgan University of Medical Sciences, Bandar Abbas, Iran; 6 Department of Pharmacognosy, Faculty of Pharmacy and Pharmaceutical Sciences, Tehran Medical Sciences, Islamic Azad University, Tehran, Iran; 7 Department of Pharmacology and Toxicology, Faculty of Pharmacy and Pharmaceutical Sciences, Tehran Medical Sciences, Islamic Azad University, Tehran, Iran; 8 Toxicology and Diseases Group (TDG), Pharmaceutical Sciences Research Center (PSRC), Tehran University of Medical Sciences (TUMS), Tehran, Iran

**Keywords:** Nephrotoxicity, Hepatotoxicity, Nasturtium officinale, Inflammation, Apoptosis

## Abstract

**Objective:**

Nasturtium officinale (N. officinale (NO)) has been widely used in traditional medicine. This study investigates the protective effects of NO against hepatic and renal damage induced by CCl_4_ and gentamicin, respectively, in rats.

**Materials and Methods:**

Male Wistar rats were divided into two arms: A (CCl4-induced hepatotoxicity) and B (gentamicin-induced nephrotoxicity). Seventeen groups were formed by dividing arms A and B, with nine groups in arm A and eight groups in arm B (n=5). Rats were daily treated with various doses (50, 100, and 200 mg/kg BW) of N. officinale extract (NOE) (Total extract; Oral gavage) for 14 and 28 days in arm A and B, respectively. Biochemical and histopathological evaluations and gene expression analyses were conducted on blood, liver, and kidney tissues.

**Results:**

NOE treatment significantly modulated B-cell lymphoma protein 2 (Bcl-2)-associated X (Bax) and B-cell lymphoma protein 2 (Bcl-2) expression in kidney tissue, reducing Bax (p<0.01) and increasing Bcl-2 (p<0.05). In liver tissue, NOE inhibited tumor necrosis factor alpha (TNF-α) (p<0.01) and Interleukin-1 beta (IL-1β) (p<0.001), while reducing AST and ALT activity (p<0.001). Additionally, blood urea nitrogen (BUN) levels significantly decreased (p<0.05) in nephrotoxic rats.

**Conclusion:**

Our findings highlight the capability of NOE as a promising therapeutic against liver and kidney damage induced by CCl_4_ and gentamicin, respectively, in animal models.

## Introduction

The liver and the kidneys are well-known organs that, by working together, maintain homeostasis in the body. The kidneys are a pair of organs that remove waste products from the blood in the form of urine and balance levels of chemical elements in the body (Hartleb and Gutkowski 2012; Raj et al. 2020). By secretion of some hormones, the kidneys check blood pressure and instigate bone marrow to produce erythrocytes (Adamson et al. 1968; Rafaqat et al. 2023). On the other hand, the liver has diverse physiological functions, including the metabolism of xenobiotics and medicines, cholesterol synthesis, and storage of micronutrients such as vitamins A, D, B_12_, and K, as well as vital minerals. Besides, the liver is responsible for the synthesis of numerous hormones, coagulation factors, immune factors, and the catalase enzyme (Hansen et al. 2020; Ramadori et al. 2008). Given these important physiological roles of both the liver and the kidneys, any defect in hepatorenal function may interrupt the stability of the body’s internal environment and, as a result, cause serious harm to the body (Calleri and Alessandria 2023). 

Specific drugs, such as antibiotics, nonsteroidal anti-inflammatory drugs (NSAIDs), immunosuppressants, chemotherapeutics, and contrast agents, are considered some of the most important factors that may cause kidney damage (Bidani and Griffin 2004). Overexpression of Transforming growth factor beta (TGF-β) and endothelin 1, increased monocyte/macrophage infiltration into the cortex and medulla of kidney, initiation of oxidative stress, apoptosis, necrosis, increased levels of serum creatinine, Blood Urea Nitrogen (BUN), albuminuria and glucosuria, and decreased levels of glomerular filtration rate (GFR) are some of the prominent events observed during nephrotoxicity (Jahnukainen et al. 2005; Lin et al. 2022). In the case of hepatotoxicity, inflammation is a part of the wound-healing process in the liver caused by harmful stimuli such as excessive alcohol and fat consumption, viral hepatitis, the presence of cholestasis, etc. It is a protective response of liver cells to a harmful stimulus that gives rise to liver tissue repair and maintains homeostasis(Leandro et al. 2006; Tanwar et al. 2020). For a limited time, inflammation may help to protect against possible liver damage; nevertheless, when inflammation becomes severe or chronic, it can lead to irreparable damage to the liver parenchyma stemming from overproduction of pro-inflammatory cytokines by non-parenchymal cells such as Kupffer cells (Koyama and Brenner 2017; Robinson et al. 2016). In this condition, acetaldehyde, reactive oxygen species (ROS), lipid peroxidation-derived compounds, and cytokines such as ILs and Tumour Necrosis Factor-alpha (TNF-α) may eventually lead to liver fibrosis (Czaja 2014). 

On the clinical scale, unfortunately, there is no approved drug to directly treat hepato- and nephrotoxicity, and treatment protocols mostly are limited to supportive care, minimizing exposure to toxic substances, and medication adjustments (Amin et al. 2019; Bera and Wong 2022). Therefore, there has been a great need to develop novel modalities to tackle this issue. 

One concept that has always been compelling to researchers in the pharmaceutical industry is to employ plant-derived compounds with satisfactory safety and efficiency that hold great promise for the development of novel medications to treat human diseases (Salmerón-Manzano et al. 2020). Indeed, medicinal plants have been used as conventional remedies to treat numerous diseases. Some herbs have hepatorenal protective properties, while others have been associated with formidable toxicity. In addition, the administration of medicinal plants as adjuvant therapy to treat hepatorenal conditions has shown interesting advantages over single-drug therapy, including lower risks of adverse effects and better prognosis in patients (Petrovska 2012; Vaou et al. 2021).

One such medicinal plant is Nasturtium officinale (NO), commonly known as watercress, an herbaceous perennial, which belongs to the Brassicaceae family and is native to Western Asia, Europe, India and Africa. Traditionally, the leaves of this plant are used to treat glycemia, lipidemia, hypertension, gastroenteritis, hypothyroidism, etc. (Al-Snafi 2020; Hibbert and Taylor 2022). Besides, NO is a diuretic and can be used in several urinary tract conditions such as kidney stones. The pharmacological effects of NO are largely due to its chemical components. Glucosinolates, isothiocyanates, and polyphenols are some of the important secondary metabolites in this plant which make it an attractive candidate in the food and cosmetic industry (Chaudhary et al. 2023; Chaudhary et al. 2018). It has been shown that the hydroalcoholic extract of the aerial parts of NO is able to decrease lipid peroxidation, glutathione levels, and mitochondrial swelling among mitochondria isolated from kidney tissue previously exposed to gentamicin (Shahani et al. 2017). Other findings show that N. officinale extract (NOE) is able to reduce serum concentrations of uric acid, malondialdehyde (MDA), and creatinine in a vancomycin-induced nephrotoxicity model. NO has also shown protective effects against liver damage via reducing the serum alkaline phosphatase(ALP), aspartate aminotransferase (AST), and alanine transaminase (ALT) activity (Al-Snafi 2020; Doustimotlagh et al. 2020). 

Considering these interesting pharmacological properties, this study aimed to investigate the protective effects of NO against hepatic and renal damage in animal models by investigating the changes in expression levels of inflammatory genes, IL-β1 and TNF-α as well as apoptotic genes, Bax and Bcl-2. 

## Materials and Methods

### Chemicals

Carbon tetrachloride (CCl_4_) and silymarin was purchased from Sigma Chemicals, USA. Gentamicin (as sulfate) was provided by Exir Co, Iran. The specific primers were purchased from Pishagam Biotech Co. Iran. TRIzol reagent was purchased from Biofact Co, China. SYBR Green qPCR Master Mix was purchased from Zistvirayesh Co, Iran. PrimeScript cDNA synthesis kit (Takara Bio Inc, Japan) was used in our investigation. Other reagents and chemicals used in this study were of analytical grade.

### Plant material and extraction

The aerial parts of N. officinale (NO) were carefully collected from Gilan province in Iran. These gathered parts were then verified by the herbarium of Tehran University of Medical Sciences (TUMS) with the voucher number: 7202-TEH, ensuring their authenticity and accuracy. Dried aerial parts of the plant were powdered and soaked with 80% methanol by maceration at room temperature. After filtration of the mixture, the filtrate was concentrated using a rotary evaporator (Heidolph, Germany) at 40ºC and low pressure and a green-colored solution was obtained. The extraction process of solution was repeated by 80% methanol until obtaining a colorless extract. After the concentration process, the NO-derived extract was poured into the crystallizer and transferred under the laboratory hood to dry up the residual solvent and a total extract was obtained.

### Animal treatment

Male Wistar rats weighing 200-250 g were provided by Faculty of Pharmacy, Tehran University of Medical Sciences. All experimental procedures used in this study were performed in accordance with the ethical standards and protocols approved by the Research Ethics Committee of Tehran Islamic Azad University of Medical Sciences - Pharmacy and Pharmaceutical Branches Faculty, Tehran, Iran with the codes of IR.IAU.PS.REC. 1402.081 and IR. IAU.PS.REC.1402.080. The minimum number of animals was employed to make a consistent effect of the treatment. For acclimatization, animals were kept in a room with suitable conditioning (humidity: 60±5%, temperature: 25±3°C) and a controlled light setting (12:12 hr light/dark cycle) for one weeks prior to the experiment. Rats were allowed free access to standard food and water during the acclimatization and experiment periods. It should be mentioned that surgical or other painful procedures were performed under appropriate anesthesia.

### Experimental design

As shown in Table 1, rats were divided into two arms (arm A: hepatotoxicity and arm B: nephrotoxicity) and then, arms A and B were subdivided into nine and eight groups, respectively. The experiment lasted for 14 and 28 days in arm A and arm B animals, respectively. At the end of mentioned study periods for each arms, animals were subjected to 24 hr of fasting after their last feeding and then, they were euthanized with overdoses of general anesthesia drugs including ketamine (100 mg/kg) and xylazine (10 mg/kg) mixture Intraperitoneal (i.p) based on standard euthanasia guidelines of animals (Wellington et al. 2013). Blood samples were collected directly from the hearts of rats for biochemical analyses. Based on previous studies, we used CCL_4_ and gentamicin as reference standards for induction of hepatotoxicity and nephrotoxicity, respectively (Mohamed et al. 2021). In addition, silymarin was used as a hepatoprotective agent and positive control in one group of arm A (Elfaky et al. 2022). 

### Biochemical analysis and liver function tests

 For biochemical studies, serum samples were obtained by centrifugation of the collected blood samples (1500 rpm, 10 min) and stored at 20°C until assayed for the biochemical parameters. Subsequently, the levels of BUN, serum creatinine (SCr), blood electrolytes (Ca, Na, and K), liver enzymes (ALT, AST and ALP) activity, and white blood cells (WBC), Red blood cells (RBC), and hemoglobin were measured using specific commercial kits by a spectrophotometric method. 

### Histopathological studies

To prepare tissues for histopathological evaluation, kidney and liver samples were placed in 10% buffered formalin. Next, the samples were transferred to 70% alcohol. Then, samples were dehydrated by alcohol with a concentration of 70% for 24 hr, 80% ethanol for 2 hr, 90%, 95% ethanol, and absolute ethanol for 20 min. Afterward, to rinse the samples, they were soaked for 20 min in Xylol I and 30 min in Xylol II. Subsequently, the samples were embedded in a paraffin block with a temperature of 56-58°C. Using a rotary microtome, the tissues were cut with a thickness of 5 μm, placed on glass that was given 70% alcohol solution, stained with hematoxylin-eosin (H&E), and finally observed under an optical microscope (Olympus, Japan).

### Gene expression study

To investigate the expression of genes Bax and Bcl-2 in the kidney tissue and TNF-α, and IL-1β genes in the liver tissue of the treated rats, real-time PCR was conducted. Prior to this experiment, tissues were kept in an RNA Later solution to prevent any possible RNA degradation. Trizol reagent was used to isolate total RNA and the cDNA was prepared with PrimeScript cDNA synthesis kits (Takara Bio Inc., Japan) based on the manufacturer’s protocol. TaKaRa LA PCR™ Kit was used to amplify cDNA fragments Real-time PCR was conducted by ABI Step One (Applied Biosystems, Sequence Detection Systems, Foster City, CA). To normalize the expression levels of our target gene according to the comparative 2 ^−ΔΔCt^ method, β-Actin was employed as the internal reference gene. Employed primers are presented in Table 2.

### Statistical analysis

The results of the present study are expressed as Mean±Standard Error of Mean (S.E.M.). One-way analysis of variance (ANOVA) and Tukey’s post hoc tests were used to analyze the statistical difference, with a p-value<0.05 considered statistically significant.

## Results

### Gene expression analysis in kidney and liver tissues

**Figure 1:**
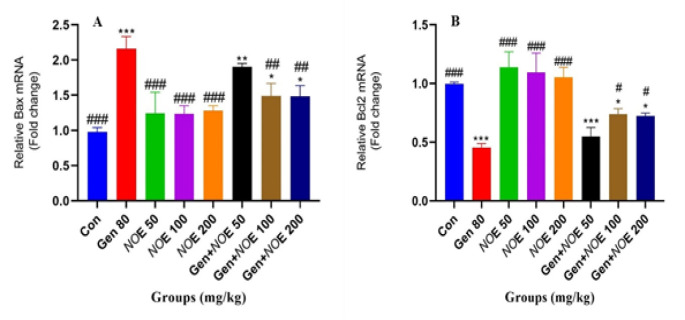
The evaluation of the expression of Bax and Bcl-2 genes in renal tissue of rats treated with Gen and the protective role of NOE; p<0.05, **p<0.01, and ***p<0.001 compared to the control group and # p<0.05, ##p<0.01, and ### p<0.001 compared to the Gen group. Abbreviation: con, control; gen, gentamicin; NOE, Nasturtium officinale extract.

As shown in Figure 1, in comparison to the control, gentamicin increased the expression levels of the Bax gene in treated rats, while it significantly reduced the expression levels of the Bcl-2 gene, a prominent anti-apoptotic gene. Although NOE at a dose of 50 mg/kg was not able to reduce the apoptotic effect of gentamicin on Bax gene expression, but at doses of 100 and 200 mg/kg, it significantly reduced the Bax gene expression compared to the gentamicin-treated group. Similarly, in the case of Bcl-2, 100 and 200 mg/kg doses of NOE were able to reverse the reduction of Bcl-2 gene expression caused by gentamicin.

**Figure 2:**
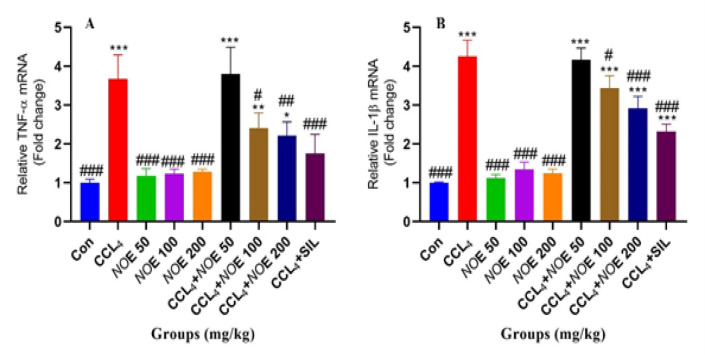
The evaluation of the expression of TNF-α and IL-1β genes in liver tissue of rats treated with CCl4 and the protective role of NOE; *p<0.05, **p<0.01, ***p<0.001 compared to the control group and #p<0.05, ##p<0.01, ### p<0.001 compared to the CCL4 group. Abbreviation: con, control; gen, gentamicin; NOE, Nasturtium officinale extract.

In hepatotoxicity models, according to Figure 2A, CCL_4_ increased TNF-α gene expression in treated rats. NOE at a dose of 50 mg/kg was not able to reduce increased TNF-α gene expression, but at doses of 100 and 200 mg/kg, it remarkably decreased the expression level of TNF-α gene compared to the CCl_4_-treated group. In addition, the expression of the IL-1β gene in the group receiving CCl_4_ (Figure 2B) was significantly increased compared to the control group. Interestingly, NOE only at doses of 100 and 200 mg/kg moderated the increased expression levels of the IL-1β gene caused by exposure to CCl_4_.

### Histopathological findings

As exhibited in Figure 3a, while no changes were observed in normal kidney tissue, microscopic observation showed significant tissue damage in the gentamicin-treated group, such as tubular changes in the form of necrosis, degeneration, and vacuolation. In addition, such changes ranged from degeneration to severe necrosis in the proximal convoluted tubules and to some extent in the distal tubules. Animals treated with NOE at a concentration of 200 mg/kg, unlike 50 and 100 mg/kg, following gentamicin injection showed tissue improvement in the glomerular structure and Bowman capsule. It is noteworthy that in groups that received NOE alone, no significant histopathological changes were observed. 

In the case of liver tissue, as shown in Figure 3b, insignificant local lesions were observed in the healthy control group, and the morphology of hepatic, sinusoidal, and trigeminal cords was normal. The negative control, which received only 1 ml/kg of CCl_4_, showed central lobular necrosis, severe loss of liver structure, and karyolysis. The group that received daily gavage of 50 mg/kg of NOE as well as CCL_4_, had a very similar structure to the negative control, and coagulative necrosis and vacuolation of liver cells were evident in most areas. The group that received 100 mg/kg of NOE and CCL_4_ had lower rates of necrosis in most parts compared to group NOE 50 mg/kg + CCl_4_ and negative control. The NOE 200 mg/kg + CCL_4_ showed the most protective effects compared to NOE 50 and 100 mg/kg. Additionally, the lowest incidence of necrosis around the portal veins was observed in this group. However, there was still some disintegration of the organization of hepatic cords and damage to sinusoids. The groups that received only oral gavage of NOE at doses of 50, 100, and 200 mg/kg were very similar to the healthy control group, and there was only a small amount of lobular center necrosis in some sections.

### Hematologic and serologic results

As shown in Tables 3 and 4, findings showed that groups treated with gentamicin (Gen) alone had higher levels of WBC, RBC, and Hemoglobin (Hb). NOE at all doses was able to rectify the increased levels of Hb, unlike WBC and RBC. In addition, the greatest reduction was observed at the dose of 200 mg/kg of NOE+Gen. The groups receiving NOE at doses of 100 and 200 mg/kg + Gen showed increased levels of BUN compared to the control group. On the other hand, the groups receiving NOE at doses of 50 and 100 mg/kg + Gen were associated with an increase in creatinine. Taken altogether, among the groups treated with Gen, the concentrations of creatinine and BUN decreased as the dose of NOE elevated. On the other hand, the levels of WBC increased in CCl_4_-treated groups, while the levels of RBC and Hb decreased. Treatment with NOE to some extent reversed the increased levels of WBC and decreased number of RBC and Hb induced by treatment with CCl_4_, indicating its potential impact on the blood profile of the examined individuals. 

Moreover, biochemical findings and liver function tests indicated that the levels of AST, ALT, ALP, SCr and BUN, which were significantly elevated in the CCl_4_-treated group, were interestingly decreased in groups treated with NOE at different doses with a dose of 200 mg/kg eliciting greatest protective effects (Tables 5 and 6).

## Discussion

Medicinal plants are known as an important source of potential compounds for developing novel drugs against various human diseases including kidney and liver diseases. Moreover, the use of medicinal plants in the pharmaceutical industry, due to their safety and efficiency, has given them significant advantages in comparison to synthetic compounds for the treatment of various diseases (Noor et al. 2022; Petrovska 2012). 

Phytochemically, N. officinale (NO) contains alkaloid compounds, flavonoids, saponins, terpenoids/steroids, proteins, volatile and essential oils, glycosides, etc. The flavonoids in NO include lutein and quercetin. Besides, NO is a rich and well-known source of benzyl glucosinolate and phenylethyl glucosinolate which are the precursors of benzyl isothiocyanate and phenylethyl isothiocyanate which have shown promising anti-cancer and anti-inflammatory effects (Al-Snafi 2020; Chaudhary et al. 2018). Several investigations have been conducted on the wide-ranging pharmacological properties of NO. Camponogara et al reported that NOE had anti-inflammatory effects in the croton oil-induced skin inflammation model. Mechanistically, NOE reduced the penetration of inflammatory cells and decreased pro-inflammatory cytokines in this inflammatory model (Camponogara et al. 2019).

One of the well-known models for establishing nephrotoxicity and evaluating the protective effects of plant-based compounds is the use of gentamicin, an aminoglycoside antibiotic, which is frequently used to combat bacterial infections caused by Gram-negative bacteria. However, its application is clinically limited due to its related nephrotoxicity (Randjelovic et al. 2017). Several investigations have indicated the involvement of oxidative stress, inflammatory and apoptotic pathways in renal damage caused by gentamicin. Natural antioxidants, various antidiabetic and antihypertensive drugs have been repurposed to counteract gentamicin-induced kidney injury in different animal models, but there is still a great need for developing novel approaches for countering this challenge with improved efficacy and safety (Balakumar et al. 2010; Walker et al. 1999). 

Bax and Bcl-2 are proteins that display a crucial role in regulating apoptosis, a kind of cell death distinguished by the activation of proteases called caspases and marked morphological alterations (Antonsson and Martinou 2000). Bax protein is a member of the Bcl-2 gene family. These proteins are involved in the mitochondrial-mediated apoptosis pathway and play a pivotal role in the balance between cell survival and death (Amini et al. 2023; Quiros et al. 2011). The effect of gentamicin on the expression of these two genes has long been proven (Servais et al. 2008). 

In our study, the effect of NOE on the expression of these two genes has been investigated following the nephrotoxicity induced by gentamicin. In addition, to better understand its protective effects, biochemical markers and histopathology of kidney tissue were put to the test. According to the results of the present study, NOE showed significant protective effects on gentamicin-induced nephrotoxicity such as modulations in the biochemical and histopathological findings as well as mRNA expression levels of Bax and Bcl-2. Indeed, at a dose of 200 mg/kg, NOE led to tissue improvements in the glomerular structure and Bowman’s capsule, along with reversing the increased levels of Hb. Moreover, this dose of NOE (200 mg/kg) reduced the expression of Bax whereas it increased the expression of Bcl2, which was remarkably different from the control group. Similar to our findings, a study conducted by Shahani et al. found that 200 mg/kg of NOE is efficient in reversing gentamicin-induced nephrotoxicity (Shahani et al. 2017). In another study conducted by Karami et al., it has been reported that the NOE with a dose of 500 mg/kg has demonstrated a protective effect against vancomycin-induced nephrotoxicity (Karami et al. 2018). Findings of a previous in vivo study exhibited that NOE with a dose of 500 mg/kg remarkably enhances the activities of antioxidant enzymes (superoxide dismutase (SOD), glutathione peroxidase (GPx), and catalase (CAT)) and reduces the content of MDA, 8-hydroxydeoxyguanosine (8-OHdG, as a biomarker of DNA damage) in kidney and liver tissues of arsenic-induced hepatorenal toxicity model. In addition, it has been reported that NOE decreases liver enzyme (ALT and AST) activities which is in line with results of our study (Zargari et al. 2014). Hosseini et al. showed the protective effects of NOE against CCl_4_-induced nephrotoxicity (Hosseini et al. 2018). According to previous studies, inhibiting the apoptotic pathway is necessary for the protective effects of NOE (Ma et al. 2021; Shahani et al. 2017). Taken altogether, our findings, which have been in concert with previous findings, propose NOE as a potential candidate against Gen-induced nephrotoxicity.

 Liver inflammation, also known as hepatitis, is a condition characterized by damage to liver cells. It can be induced by multiple factors including heavy alcohol consumption, viral diseases (such as hepatitis A, B, and C), toxins, certain pharmaceutical drugs, and autoimmune disorders. Immune response and inflammatory processes play a key role in the emergence and progression of liver diseases and make the regulation of inflammation a key target for therapeutic strategies (Bishayee 2014). Carbon tetrachloride (CCl_4_) is another compound that is commonly employed to establish hepatotoxicity and evaluate the protective effects of different plant-based derivatives. Various studies have shown that CCl_4_ can cause liver inflammation and injury and lead to marked fibrosis and other liver-related diseases (El-Kot et al. 2023; Gupta et al. 2022). On the other hand, silymarin, a widely-used bioactive mixture derived from Silybum marianum (milk thistle), that serves as an effective antioxidant, antifibrotic, and anti-inflammatory agent against chemical-induced hepatotoxicity in animal models (Vargas-Mendoza et al. 2014). 

AST and ALT are enzymes released by the liver upon injury and/or inflammation. They are important markers for liver function and inflammation, and their levels can indicate the presence of liver damage or disease. These enzymes are used as indicators of liver function. An increase in liver enzymes often indicates inflammation or damage to liver cells (Gowda et al. 2009; Kasarala and Tillmann 2016). Liver inflammation and fibrosis are regulated by complex immunological pathways, and pro-inflammatory cytokines, such as TNF-α, IL-1 and IL-6. TNF-α triggers Kupffer cells and macrophages to express the inflammatory mediators IL-1β, IL-6, and major fibrogenic cytokines (Campana et al. 2021; Manns et al. 2010). Similar to the previous section, findings highlighted that NOE, especially at a dose of 200 mg/kg, significantly modulated the expression of the inflammatory genes; it decreased the expression levels of TNF-α and IL-1β genes compared to the CCl_4_-treated group, suggesting its potential hepatoprotective effects. Likewise, histopathological findings showed the lowest incidence of necrosis following the administration of a 200 mg/kg dose of NOE. Moreover, biochemical findings indicated that the levels of AST, ALT, ALP, SCr, and BUN, which were previously increased in the CCl_4_-treated group, were protectively decreased in groups treated with NOE, especially at a dose of 200 mg/kg. In line with these results, some studies have also found that NOE reduced hepatotoxicity by decreasing expression levels of pro-oxidants and interleukins (Hossini et al. 2017; Rostam et al. 2021). Besides, a study by Wang et al. showed that the oral ethanol extract of Lonicera japonica against liver showed hepatoprotective effects against damage caused by CCl_4_ in rats via decreasing the activity of liver enzymes AST, ALT, and ALP (Wang et al. 2022). All in all, these findings once again put forward NOE as a promising candidate with hepatoprotective effects.

With promising findings about the protective effects of NOE against hepatorenal damage, however, our study, like many others, faces certain limitations that call for further investigations in the future. Indeed, most NOE studies regarding hepatorenal damage are only limited to preclinical models and lack any significant data at the clinical scale (Al-Snafi 2020). Besides, most studies in this regard mainly evaluated the short-term effects of NOE on acute hepatorenal damage whereas only a disproportionate number focused on its long-term use (Shekarforoush et al. 2016), which indeed warrants more investigations on long-term effects of NOE as well as any pernicious side effects. Moreover, because NOE contains several bioactive compounds, there is a potential risk of unwanted interactions between NOE and other co-administered drugs which can alter the drug metabolism and transport and, overall, compromise the efficacy of the treatment (Gómez-Garduño et al. 2022). Given that, future studies need to overcome such limitations and help researchers better understand the protective effects of NOE against hepatorenal damage.

In conclusion, the use of medicinal plants, such as NOE, shows promise in protecting against hepatorenal damage. This study demonstrated the protective effects of NOE against liver and kidney damage induced by CCl_4_ and gentamicin, respectively, in animal models. The extract resulted in modulation of mRNA expression levels of some inflammatory and apoptotic genes, as well as changes in histopathological and biochemical markers all in favor of normal functions of the liver and kidney. With safer and more efficacious impact, plant-derived compounds hold great promise for future clinical translation which necessitates further investigations regarding this matter. 

**Table 1:** Experimental designs for evaluation of Nasturtium officinale extract (NOE) effect on hepatotoxicity (Arm A) and nephrotoxicity (Arm B).

**Group No.**	**Arm A (n= 45; 5 rats per each group)**	**Arm B (n=40; 5 rats per each group)**
Group 1	Con: healthy rats with no treatment	Con: healthy rats with no treatment
Group 2	CCl_4_: rats were treated with CCl_4_ dissolved in olive oil 1 mg/kg BW, i.p., two times, on the 7^th^ and 14^th^ days)	Gen 80: rats were treated with gentamicin 80 mg/kg BW, i.p., twice a week, for four weeks)
Group 3	NOE 50: rats were treated daily with 50 mg/kg BW of Nasturtium officinale extract (NOE) by gavage for two weeks.	NOE 50: rats were treated daily with 50 mg/kg BW of Nasturtium officinale extract (NOE) by gavage for four weeks.
Group 4	NOE 100: rats were treated daily with 100 mg/kg BW of Nasturtium officinale extract (NOE) by gavage for two weeks.	NOE 100: rats were treated daily with 100 mg/kg BW of Nasturtium officinale extract (NOE) by gavage for four weeks.
Group 5	NOE 200: rats were treated daily with 200 mg/kg BW of Nasturtium officinale extract (NOE) by gavage for two weeks.	NOE 200: rats were treated daily with 200 mg/kg BW of Nasturtium officinale extract (NOE) by gavage for four weeks.
Group 6	CCl_4 _+ NOE 50: rats were treated daily with 50 mg/kg BW of Nasturtium officinale extract (NOE) by gavage for two weeks and were injected with CCl_4_ dissolved in olive oil (1 mg/kg BW, i.p., two times, on the 7^th^ and 14^th^ days)	Gen + NOE 50: rats were treated daily with 50 mg/kg BW of Nasturtium officinale extract (NOE) by gavage for four weeks and were injected with gentamicin (80 mg/kg BW, i.p., twice a week, for four weeks)
Group 7	CCl_4 _+ NOE 100: rats were treated daily with 100 mg/kg BW of Nasturtium officinale extract (NOE) by gavage for two weeks and were injected with CCl_4_ dissolved in olive oil (1 mg/kg BW, i.p., two times, on the 7^th^ and 14^th^ days)	Gen + NOE 100: rats were treated daily with 100 mg/kg BW of Nasturtium officinale extract (NOE) by gavage for four weeks and were injected with gentamicin (80 mg/kg BW, i.p., twice a week, for four weeks)
Group 8	CCl_4 _+ NOE 200: rats were treated daily with 200 mg/kg BW of Nasturtium officinale extract (NOE) by gavage for two weeks and were injected with CCl_4_ dissolved in olive oil (1 mg/kg BW, i.p., two times, on the 7^th^ and 14^th^ days)	Gen + NOE 200: rats were treated daily with 200 mg/kg BW of Nasturtium officinale extract (NOE) by gavage for four weeks and were injected with gentamicin (80 mg/kg BW, i.p., twice a week, for four weeks)
Group 9	CCl_4 _+ SIL (positive control): rats were treated daily with 40 mg/kg BW of silymarin by gavage for two weeks and were injected with CCl_4_ dissolved in olive oil (1 mg/kg BW, i.p., two times, on the 7^th^ and 14^th^ days)	-

Abbreviation: Con, Control; i.p, Intraperitoneal; Gen, gentamicin; BW, Body Weight; NOE, Nasturtium officinale extract .

**Table 2:** The List of the employed primers

**Gene**	**Primer (Oligo Sequence 5'--> 3')**
Bax	CAACCTTCTTGCAGCTCCTC (F)
	CTTTCCCCGTTCCCCATTCA (R)
Bcl-2	TCGCGACTTTGCAGAGATGT (F)
	CAATCCTCCCCCAGTTCACC (R)
TNF-α	ACTGAACTTCGGGGTGATCG (F)
	GCTTGGTGGTTTGCTACGAC (R)
IL-1β	TTGAGTCTGCACAGTTCCCC (F)
	GTCCTGGGGAAGGCATTAGG (R)
β-Actin	CAACCTTCTTGCAGCTCCTC (F)
	TTCTGACCCATACCCACCAT (R)

**Figure 3a:**
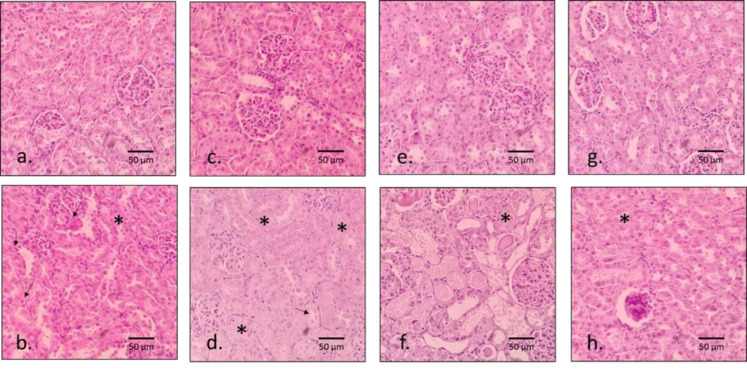
The histological examination (H&E staining) (200x) of the kidneys. The arrow indicates the degeneration of renal tubules, whereas the star indicates the degeneration and necrosis of renal tubules. (a): healthy group, (b): negative control group (Gentamicin-treated rats), (c): healthy group treated with NOE 50 mg/kg BW , (d): animal group exposed to NOE 50 mg/kg BW, (e): healthy group treated with NOE 100 mg/kg BW, (f): animal group exposed to NOE 100 mg/kg BW, (g): healthy group treated with NOE 200 mg/kg BW, and (h): animal group exposed to NOE 200 mg/kg BW.

**Figure 3b:**
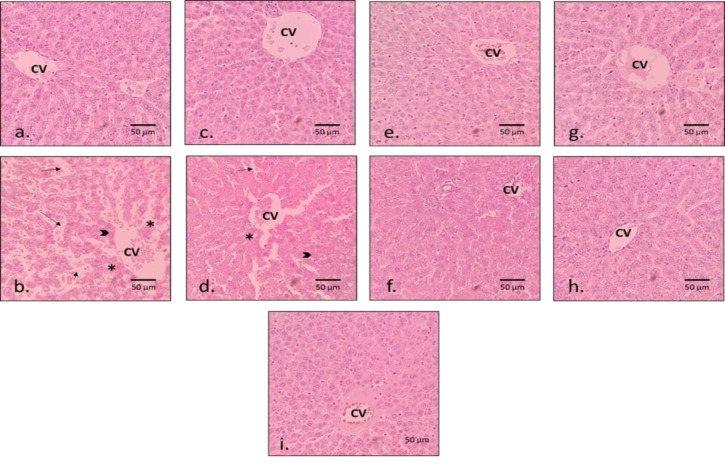
H &E staining of the histopathological sections of the liver; the stars indicate necrosis, the arrowheads indicate karyolysis, and the pointing arrows indicate disarrangement of liver fibers. (a): healthy (control) group, (b): negative control group (CCl4-treated rats), (c): healthy group with NOE 50, (d): exposed animal group with NOE 50 mg/kg BW, (e): healthy group with NOE 100 mg/kg BW, (f): exposed animal group with NOE 100 mg/kg BW, (g): healthy group with NOE 200 mg/kg BW, (h): exposed animal group with NOE 200 mg/kg BW, and (i): positive control group (CCL4+silymarin-treated group). Abbreviations: CV: central vein.

**Table 3:** Hematological parameters of male Wistar rats after treatment with gentamicin and Nasturtium officinale extract (NOE).

**Groups**	**Hematological parameters**		
	**WBC** (**1000/μl**)	**RBC (mil/ul)**	**Hb ** **(g/dl)**
Group 1 (Con)	8.36±2.24	10.15±1.27	10.22±2.34
Group 2 (Gen 80)	10.24±3.23	9.51±1.24	6.41±1.34**
Group 3 (NOE 50)	9.17±1.49	9.46±1.19	9.24±2.11
Group 4 (NOE 100)	9.27±1.25	10.38±1.15	9.27±2.18
Group 5 (NOE 200)	9.36±2.19	9.27±2.22	10.53±2.24
Group 6 (Gen+NOE 50)	8.23±1.65	10.54±0.12	6.24±1.37**
Group 7 (Gen+NOE 100)	9.32±2.17	10.26±1.84	7.15±2.3*
Group 8 (Gen+NOE 200)	8.54±1.23	10.25±2.67	8.47±1.15

Con, Control; Gen, Gentamicin, NOE, Nasturtium officinale extract; WBC, White blood cells; RBC, Red blood cells, Hb, Hemoglobin. p ≤ 0. 01 ** and p ≤ 0.05* are considered significant changes in relation to control.

**Table 4:** Hematological parameters of male Wistar rats after treatment with CCL4, silymarin and Nasturtium officinale extract (NOE).

**Groups**	**Hematological parameters**		
	**WBC** (**1000/μl**)	**RBC (mil/ul)**	**Hb ** **(g/dl)**
Group 1 (Con)	8.42±1.12	12.31±2.35	11.15±1.17
Group 2 (CCl_4_)	12.73±1.08*	6.42±5.58**	6.2±0.72*
Group 3 (NOE 50)	9.11±2.04	10.15±0.12	9.47±1.53
Group 4 (NOE 100)	8.11±1.06	11.15±0.06	10.36±1.12
Group 5 (NOE 200)	10.21±2.93	10.16±1.23	9.72±2.1
Group 6 (CCl_4 _+NOE 50)	13.42±2.32*	7.11±2.36*	6.18±2.44*
Group 7 (CCl_4 _+NOE 100)	12.39±1.50*	9.28±2.3	10.2±1.4
Group 8 (CCl_4 _+NOE 200)	12.10±1.37*	9.18±1.17	12.41±2.53
Group 9 (CCl_4 _+ SIL)	10.44±1.8	10.31±0.52	10.32±2.51

Con, Control; SIL, Silymarin, NOE, Nasturtium officinale extract; WBC, White blood cells; RBC, Red blood cells, Hb, Hemoglobin. p≤0. 01 ** and p≤0.05* are considered significant changes in relation to control.

**Table 5:** Biochemical results of blood serum samples in male Wistar rats after treatment with gentamicin and Nasturtium officinale extract (NOE).

**Groups**	**Biochemical parameters of serum**	
	**BUN** **(mg/dl)**	**SCr (mg/dl)**
Group 1 (Con)	66.25±3.15	0.63±0.08
Group 2 (Gen 80)	101.1±10.42***	0.94±0.03**
Group 3 (NOE 50)	65.31±2.17	0.69±0.04
Group 4 (NOE 100)	67.18±3.24	0.67±0.03
Group 5 (NOE 200)	69.12±1.38	0.62±0.03
Group 6 (Gen+NOE 50)	96.31±8.23**	0.91±0.04**
Group 7 (Gen+NOE 100)	91.73±5.41**	0.83±0.02*
Group 8 (Gen+NOE 200)	80.71±6.24*^,#^	0.80±0.03*

Con, Control; Gen, Gentamicin, NOE, Nasturtium officinale extract; BUN, Blood urea nitrogen; SCr, Serum creatinine. p≤0.01 ** and p≤ 0.05* are considered significant changes in relation to control. p≤0.05# is considered a significant change between Group 8 and Group 2.

**Table 6:** Biochemical results of blood serum samples and results of liver functions tests in male Wistar rats after treatment with CCL4, silymarin and Nasturtium officinale extract (NOE).

**Groups**	**Hematological parameters**							
	**BUN** **(mg/dl)**	**SCr ** **(mg/dl)**	**Ca** **(mg/dl)**	**Na** **(mEq/L)**	**K** **(mEq/L)**	**AST** **(U/L)**	**ALT** **(U/L)**	**ALP** **(U/L)**
Group 1 (Con)	67.21±2.16	0.74±0.09	10.53±0.11	136.22±1.04	4.39±0.48	171±8.12	75±1.24	411.21±164.21
Group 2 (CCl_4_)	97.26±4.2***	0.85±0.03	13.51±0.12	141.52±2.27	4.19±0.66	541±8.63***	208.7±1.24***	1121.27±201.34***
Group 3 (NOE 50)	66±6.52	0.67±0.02	0.67±0.02	134.23±1.52	3.25±0.14	175.3±11.24	71.3±1.12	422.51±142.11
Group 4 (NOE 100)	67.15±7.12	0.66±0.02	11.31±0.14	138.11±1.12	4.7±0.49	182.35±24.41	73.2±2.41	501.29±86.28
Group 5 (NOE 200)	70.15±2.11	0.62±0.03	11.27±0.74	137.15±1.37	4.31±0.03	183.27±18.71	69.1±1.18	508.42±141.27
Group 6 (CCl_4 _+NOE 50)	89.32±4.13**	0.59±0.08	12.78±0.31	140.54±0.43	4.74±0.3	421.33±41.27***	182.5±2.14***	1267±96.04***
Group 7 (CCl_4 _+NOE 100)	86.11±7.25**	0.73±0.6	10.89±0.39	141.18±2.11	4.71±0.38	401.17±26.14***	199.1±4.36***	1093.52±147.23***
Group 8 (CCl_4 _+NOE 200)	79.28±8.68*	0.78±0.04	11.47±0.41	139.21±2.14	4.72±0.61	315.33±51.25**^,###^	151±6.32**^,###^	987.11±201.14**
Group 9 (CCl_4 _+ SIL)	81.27±2.11*	0.81±0.03	12.30±0.45	140.13±1.14	4.07±0.68	308.23±41.39**	136.2±1.23**	811.41±159.12**

Con, Control; SIL, Silymarin, NOE, Nasturtium officinale extract; BUN, Blood urea nitrogen; SCr, Serum creatinine; AST, Aspartate aminotransferase, ALT, Alanine transaminase; ALP, Alkaline phosphatase. p≤0.01 **, p≤0.001 *** and p≤0.05* are considered significant changes in relation to control. p≤0.001 ### is considered a significant change between Group 8 and Group 2.
